# Ichthyol in Skin Diseases

**Published:** 1886-12

**Authors:** Joseph Zeisler

**Affiliations:** 125 State Street


### The Use of Ichthyol in the Treatment of Skin Diseases.

*By* Joseph Zeisler, M.D.

[Read before the Chicago Medical Society, November 15th, 1886.]

Only a few years ago ichthyol was introduced into dermatological practice by that industrious worker Unna, in Hamburg, to whom we owe so many other practical contributions. The question of its merits was discussed by a number of writers, some praising it enthusiastically, others attributing very little value to it. As to my own experience, the results obtained by me in using this drug for nearly one and a half years were so favorable that I deem it my duty to give an account of the same, and in so doing contribute toward a better knowledge of that valuable remedy. It is not the object of this paper to enter into a discussion of the chemical properties of ichthyol, or, as it is properly called, sulpho-ichthyolate of ammonia. Nor shall I dwell at length on its physiological action upon the normal and diseased skin; this was done in a very extensive manner in Unna's last paper on the subject*, an abstract of which appeared in the *Journal of Cutaneous and Venereal Diseases* of September last. To anyone who is specially interested in the subject, a careful perusal of the

* Ichthyol and Resorcin. Leipzig and Hamburg, 1886.

original will be of the greatest advantage. My own attention was first attracted to ichthyol by a short communication in the *Deutsche Mediz. Wochenschrift, 1885*, wherein Lorenz speaks in the highest terms of its wonderful action in articular rheumatism, when used externally over the inflamed joints. Only short reference was made there to its power of arresting inflammation and relieving local pains. Since then I used it in over 100 cases of skin diseases, and without claiming for it any specific effect, I may say that I owe some of my best results to it, and that I very seldom was disappointed by the same.

In looking over my record, I find that ichthyol was prescribed in the following cases: Eczema, 56; acne vulgaris, 10; acne rosacea, 7; sycosis, 11; herpes tonsurans, 4; telangiectasis, 4; prurigo, 2; pruritus, 2; psoriasis vulg., 2; psoriasis palmaris specifica, 2 cases; seborrhœa, acne varioliformis, lichen pilaris, impetigo contagiosa, purpura rheumatica and ulcera cruris, each 1 case. In some of these it was only used as an adjuvant. In all of them I applied it externally, in the form of salve, the strength varying from 3 to 30 per centum. It mixes very well with all of the ointment-bases; as such, were used mostly vaseline, cold-cream, zinc salve and lanolin, to which were sometimes added other drugs, as adjuvants, like salicylic acid, præcip. album, resorcin, naphtol, sapo viridis and others, according to the requirements of the case. The rather strong and peculiar smell of ichthyol, although not very offensive, can easily be overcome when used in the form of ointments. A slight brownish discoloration following its use will usually soon disappear. No other inconvenience was noticed from its external application, except in a few instances where a slight burning sensation was complained of, especially when used on the face. Several times I observed that soon after the application of an ichthyol compound the skin would be covered with a watery fluid on the anointed parts, like little dew-drops. Lorenz, who seems to have made the same observation, interprets it as hyperidrosis. Unna very often prescribes ichthyol in watery solution and in the form of a paste. I have so far made no use of these forms, but I think that where only superficial action is desired it may be preferable to ointments for technical reasons. For many cases, particularly acne sycosis and acne rosacea, I found ichthyol soap very convenient. In this form it may be used either like any other toilet soap, or the foam of it may be allowed to remain on the skin for a short while, or even for many hours, according to the effect desired. The physiological effect of ichthyol, when used externally, can be inferred from its chief quality—that is, to draw oxygen from the tissues. Unna therefore calls it a reducing agent, and places it in the same class with resorcin, pyrogallol and chrysarobin. Its regenerative power on one hand, when used in a mild form; its resolving action on the other hand, when used in full strength; its contracting influence upon blood-vessels, can easily be explained from that one principal quality.

Upon the suggestion of Unna, I have in the last five months prescribed ichthyol also for internal use in some twenty-five cases of chronic skin diseases, with apparently very satisfactory result. At this place I cannot resist the temptation to offer a few remarks in regard to the internal treatment of cutaneous diseases, as this question has recently been much discussed. As is well known, the Vienna school very seldom resorts to it, while American physicians, from their somewhat different ætiological standpoint, mostly depend upon the internal administration of certain drugs, claimed to remove so-called impurities of the blood. Without discussing this fundamental question here as thoroughly as it would deserve, I may say that I believe in a reasonable internal treatment adapted to the merits of each case, inasmuch as sometimes disorders of internal organs seem to have a causal relation to that of the skin. But the daily routine practice of administering arsenic indiscriminately, even without a proper diagnosis, cannot be too strongly repudiated. In a recent article * G. H. Fox made very timely remarks upon this subject, denouncing in strong terms the injudicious use of arsenic. I heartily concur in his views, and would only add that the same have been fully endorsed by many authorities in this country. While arsenic may have even specific effects in lichen ruber and psoriasis, and may be very useful in some other chronic cases of skin diseases, it is, on the other hand, very often without any effect, but perhaps still oftener aggravates the condition which it was intended to allay. I foresee a far wider field for ichthyol. A number of observations on the excellent results obtained by its use in cases of chronic dyspepsia and rheumatism are already published, and I can add some more made in my own practice. It is therefore obvious that wherever the last mentioned affections appear as complications or causes of cutaneous diseases, the latter will indirectly be beneficially influenced by the internal use of ichthyol. It is as yet rather difficult to determine in what way ichthyol taken internally is capable of affecting the skin itself. Clinical observations, however, make it highly probable that it has a contracting effect upon enlarged blood-vessels of the same, as can be seen in cases of vaso-motor neuroses, e. g. acne rosacea; in such cases

* N. Y. Medical Monthly, No. 1.

especially it is recommended by Unna. Private practice affords very little opportunity to make experiments with new methods or new drugs; I am therefore at a loss to state how much of the result obtained in each case was due to the internal medication, as always topical treatment was mostly relied upon. But in several instances I could clearly trace the disappearance of a chronic catarrh of the stomach or a rheumatic affection to ichthyol. Unna also reports good results in cases of asthma and of general mal-nutrition. Unlike arsenic, ichthyol will never be found to molest even a very delicate stomach, though given in doses up to 1.00 (15 gr.) a day; in fact, my patients never would feel any bad effect whatever from its use. I usually prescribe it in form of capsules, each containing 0.10 (1½ gr.), three to ten of which are taken daily after meals. For children, or patients who object to pills, a liquid form will be more convenient. The dose for infants will of course be comparatively smaller.

The fifty-six cases of *eczema*, in which I used ichthyol comprise nearly all forms and stages of that protean disease. Unlike tar, it can be very well applied even to weeping surfaces, which will sometimes get dry in one or two days; small vesicles and pustules will disappear much more promptly than by any other treatment. Its reducing power will best be seen in those hard, infiltrated patches that resist so obstinately the usual methods; they will sometimes get paler and softer within a few days. Itching, which in such cases is almost unbearable, will often be promptly relieved by it. Its regenerative power will, on the other hand, be noticed in cases of chronic eczema of the palms and fingers, where even deep fissures will heal up in short time under its application. In two cases of intertrigo on infants, where different methods had done no good, a 5 per centum ichthyol salve had an almost magical effect after one day. I mention this especially, because Stelwagon, in a note on ichthyol,* says that it is irritating in erythematous eczema. As this writer seems to have seen no positively beneficial effect in the twelve cases of eczema where he tried it, I would like to state here my experience, as follows: In the above mentioned fifty-six cases the effect was excellent in eleven, good in twenty-seven, fair in four, negative in one, and unknown in thirteen cases. The strength used was, on the head and face, from 5 to 10 per centum; on other parts of the body, usually from 10 to 30 per centum; in erythematous forms it should not exceed 5 per centum. Zinc ointment was mostly used as a base. An addition of 5 to 10 per centum salicylic acid often seemed to increase the efficacy in old, infiltrated places. I will not weary you with many illustrations; allow me to cite just two.

I. Mr. E. F. H., 49 years, merchant, had been suffering from chronic eczema for twelve years; it mostly affected the hands and the anus. Chronic dyspepsia; otherwise healthy. When I first saw him his disease was in a very aggravated condition, and presented the following appearance: June 13th, 1886. The entire right hand was considerably swollen, reddened and painful; covered all over with vesicles in all stages of development; some larger blebs filled with purulent contents; eczema vesiculosum of the left hand in minor degree; eczema erythematosum of the neck; eczema madidans on forehead, chin and scrotum. Zinc ointment with an addition of 10 per centum ichthyol was applied to all the affected parts, except the right hand, for which cold applications of thymol 1:1000 were used, by which the pain and swelling were diminished after two days;

* Note on drugs. Journal of C. and V. Dis., November, 1886.

ichthyol salve was then likewise used here. The skin all over the body was so irritable that slight scratching would suffice to produce an erythematous or even a bullous eruption. But the use of the ichthyol salve would usually cause the latter to disappear in one or two days.

June 18th. No new blisters coming out on the right hand; pain, redness and swelling considerably decreased; the smaller vesicles mostly dried, whereas the larger ones became denuded. All other affected spots are nearly healed, except that a slight scaling has set in.

June 22d. All the open places on the right hand are covered with new epidermis, while the old skin peals off abundantly. Ichthyol internally, 0.30 *pro die.*

June 28th. Slight scaling on the formerly affected parts. Patient feels very comfortable. Ichthyol capsules, 0.60 daily.

August 23d. Patient has felt excellently during the last two months. His dyspepsia does not trouble him. He assures me that he never was so rapidly cured of any of the outbreaks of his disease.

October 13th. Patient has continued to take ichthyol internally up to now, and feels first rate. There is no sign of his former trouble.

II. Rev. M. N. Chronic eczema of the right lower leg for four years. Twenty-three physicians have been consulted during this time without favorable result.

Status praes. February 13, 1886. The lower part of the right leg very much reddened, somewhat swollen, painful and itchy; covered with small pustules. An examination of the hairs forming the center of these pustules showed them to be in a similar condition as is found in sycosis. The treatment consisted in epilation of most of the affected hairs and application of the following salve: cold-cream, 40.00; ichthyol, 4.00; acid. salicyl., 2.00. No internal treatment.

February 19. The pustules have disappeared, the skin generally paler, no pain, little itching. Prescribed a 15 per centum ichthyol salve, similar to the first.

March 5, when the patient was seen for the fourth time, no abnormity could be noticed except a slight redness of the skin. The hairs extracted apparently sound.

I lately heard from the patient, who has been all right since.

*Sycosis.*—All of the above mentioned eleven cases were rather severe and inveterate, so that epilation could not be dispensed with. This method and the use of ichthyol soap and a 10 per centum ichthyol ointment had an excellent effect in four, a good one in seven cases after one to three weeks. The combination with tar-oil and overfatted green soap, recommended by Unna, seemed to me to have an irritating effect.

In *acne vulgaris* I did not notice any remarkable influence of ichthyol, until I used it internally, when it seemed to have a decidedly beneficial effect in three cases.

In *acne rosacea* it worked very satisfactorily in five, remarkably well in two cases. One of these two, the wife of a well-known physician of this city, had suffered from the disease for several years and presented, when I first saw her, the typical picture of the more severe form of acne rosacea. Besides a 10 per centum ichthyol-salve and sulphur-naphtol soap, mechanical treatment was made use of from time to time. After several weeks a decided improvement was noticeable. The lady has now been under my observation for nearly one year, and during this time has gradually improved until now, when recovery seems almost perfect. No internal medication whatever had been used; only of late ichthyol pills were recommended to check a possible relapse.

A similar success was obtained in the other case of a gentleman—a prominent member of the legal profession—who for many years was suffering from that disease. He came under my observation in October, 1885, and was cured after six weeks. He had no relapse during the last year.

In three out of the four cases of *herpes tonsurans* a combination of equal parts of oil of cade, green soap, ichthyol and vaseline had a very decidedly positive effect. In one of them a cure was effected within one week, after many remedies had been tried in vain.

In four cases of small circumscribed *telangiectasis* in the face (so-called rosacea) it had no effect; I suppose because the salve used was too mild to exert a contracting effect upon the ectatic blood vessels.

In *prurigo* it was used combined with naphthol; the effect was very good in the above two cases.

In *psoriasis* ichthyol was only used intermediately, to alleviate the inflammation produced by chrysarobin. In one case, where arsenic could not be given, the ichthyol pills seemed to work very nicely.

In regard to the rest of the above mentioned cases, I would not like to draw any conclusions, because of the small number of observations. Only one case seems to me worth mentioning; that is one of *acne varioliformis.*

A gentleman of thirty-eight had suffered for two years from repeated outbreaks of that rather rare and troublesome affection, which had left numerous scars on the scalp and forehead. In this case a 10 per centum ichthyol salve, with an addition of 5 per centum hydrarg. ammoniat., made the fresh pustules dry up within four days, and the remaining scars were very insignificant. From a continued use, the whole scalp, formerly very red, got paler, and even the old scars were much less visible.

It is a question of time and continued observations to clearly define the limits in which ichthyol will find its useful application. I hardly think that it will have to share the fate of so many other new drugs, which at first found their enthusiastic admirers, and, after considerable disappointment, sank into oblivion; but I believe, confidently, that it will gain a permanent place in dermatological therapeutics. I am only sorry that a man of Piffard's reputation unjustly places ichthyol in the same rank with cuticura and similar proprietary compounds, because, unfortunately, some enterprising firm in New York seems to have extensively advertised it.* In this city, at least, quackery does not seem to have taken hold of the new remedy. As far as I am concerned, I would by no means, after my experience, like to miss ichthyol from my armamentarium.

125 State Street.

---

* Note on drugs.—Jour. of C. and V. Dis. No. X., 86.

